# Short-Term Influence of Caffeine and Medium-Chain Triglycerides on Ketogenesis: A Controlled Double-Blind Intervention Study

**DOI:** 10.1155/2021/1861567

**Published:** 2021-06-15

**Authors:** Anna Baumeister, Joachim Gardemann, Manfred Fobker, Verena Spiegler, Tobias Fischer

**Affiliations:** ^1^University Hospital Muenster, Department of Pediatrics, Albert-Schweitzer-Campus 1, Münster 48149, Germany; ^2^University of Applied Sciences, Department of Food, Nutrition,and Facilities, Corrensstraße 25, Münster 48149, Germany; ^3^University of Muenster, Institute of Pharmaceutical Biology and Phytochemistry, Corrensstraße 48, Münster 48149, Germany

## Abstract

**Background:**

Ketone bodies are a highly relevant topic in nutrition and medicine. The influence of medium-chain triglycerides (MCT) on ketogenesis is well known and has been successfully used in ketogenic diets for many years. Nevertheless, the effects of MCTs and coconut oil on the production of ketone bodies have only partially been investigated. Furthermore, the increased mobilisation of free fatty acids and release of catabolic hormones by caffeine suggest an influence of caffeine on ketogenesis.

**Methods:**

In a controlled, double-blind intervention study, seven young healthy subjects received 10 mL of tricaprylin (C8), tricaprin (C10), C8/C10 (50% C8, 50% C10), or coconut oil with or without 150 mg of caffeine, in 250 mL of decaffeinated coffee, over ten interventions. At baseline and after every 40 minutes, for 4 h, *ß*HB and glucose in capillary blood as well as caffeine in saliva were measured. Furthermore, questionnaires were used to survey sensory properties, side effects, and awareness of hunger and satiety.

**Results:**

The interventions with caffeine caused an increase in *ß*HB levels—in particular, the interventions with C8 highly impacted ketogenesis. The effect decreased with increased chain lengths. All interventions showed a continuous increase in hunger and diminishing satiety. Mild side effects (total = 12) occurred during the interventions.

**Conclusions:**

The present study demonstrated an influence of caffeine and MCT on ketogenesis. The addition of caffeine showed an additive effect on the ketogenic potential of MCT and coconut oil. C8 showed the highest ketogenicity.

## 1. Introduction

Coffee has been a popular beverage worldwide for centuries. There are many reasons for coffee consumption, such as social aspects, wellbeing, enjoyment, and with increasing relevant positive health effects [1, 2]. The main active ingredient in coffee is caffeine, a trimethylated xanthine derivate belonging to the group of alkaloids [3]. Caffeine has a high bioavailability of nearly 100% and is mainly degraded by cytochrome P450 in the liver. The half-life of caffeine is 2.5–4.5 h [4]. Caffeine has a stimulating effect on the central nervous system, which is associated with a release of catecholamines [5, 6]. An increase in thermogenesis was described as early as 1915 and was confirmed in later studies [7–10]. In this context, an increased lipolysis and the release of free fatty acids were also described [10–13]. In addition to its influence on energy balance, coffee also has antioxidant, anti-inflammatory, antidiabetic, and other effects on health [2, 14, 15].

In association with the health effects of coffee, the trend beverage “bulletproof coffee” is under scrutiny. The development of bulletproof coffee goes back to an American biohacker who claims that the drink increases energy, performance, and satiety [16]. The composition of bulletproof coffee varies; in most cases, it is a mixture of coffee, pasture butter, coconut oil, and medium-chain triglycerides (MCTs). The addition of only MCTs in coffee is also frequently used for bulletproof coffee. However, the effects described so far have been insufficiently investigated and may instead be caused by caffeine and a stimulation of ketogenesis by fats, especially MCTs.

Ketogenesis occurs during starvation, intense fasting, or prolonged physical exertion and is thus important for supplying energy in the absence of carbohydrates. The most important quantitative ketone body is *ß*-hydroxybutyrate (*ß*HB), followed by acetoacetate [17]. Under regular conditions, ketone bodies are present in blood only at low levels (<0.1 mmol/L) [18, 19]. After a fasting period of 24–36 hours, *ß*HB concentrations of 2-3 mmol/L are reached, which can rise to 6–8 mmol/L if starvation persists [17]. Ketogenesis may also be stimulated by ketogenic diets, the main application of which is in treating pharmacoresistant epilepsy in children and adolescents. However, these diets have also been successfully used in the therapy of some inborn metabolic errors [20]. The diets are based on a very high-fat diet with simultaneous carbohydrate restriction, whereby the metabolism is switched to fat and ketone body utilisation [21].

Especially in ketogenic diets, MCTs have a special role and are often described as boosters. Due to rapid absorption, distribution, and preferential oxidation, MCTs produce a higher formation rate of ketone bodies. As such, MCTs can be used to stimulate and/or stabilise ketone-body levels during a ketogenic diet and increase compliance [22–24]. Courchesne-Loyer et al. showed that MCTs induce mild ketosis even in the absence of fasting or ketogenic diets [25]. Apart from in clinical applications, there is an increasing evidence that MCTs and ketone bodies have other positive effects, such as increased satiety, neuroprotective effects, and improved memory [26, 27].

Studies investigating the influence of caffeine and MCTs of different chain lengths on ketogenesis are currently insufficient. It is therefore impossible to determine the benefits of food trends and the use of MCTs of different chain lengths and caffeine in clinical diets. This study therefore investigates the influence of caffeine and different MCT products on ketogenesis using a minimally invasive kinetics study as well as the influence of such products on hunger and satiety.

## 2. Methods

### 2.1. Study Population

The criteria for inclusion were the absence of metabolic diseases (such as diabetes), the absence of other chronic or acute diseases (including psychological disorders), nonsmoker, not pregnant, normal BMI, no intolerance to MCT or caffeine, no special nutrition habits (such as low carb, paleo, vegan, or ketogenic), and no drug use (excluding oral contraceptives) or use of nutritional supplements in the 30 days prior to the start of the study. Participants were excluded if they became ill before or during the study. Recruitment was done using an online questionnaire (QuestionPro®), after which an informative conversation with qualified participants, ideally resulting in written consent, took place. The participants were then asked to avoid eating and drinking (except for water) for 12 hours before testing, and for 24 hours for alcoholic and caffeinated beverages, and then to arrive with an empty stomach. The study was conducted in accordance with the Declaration of Helsinki, and the protocol was approved by the Ethics Committee of the medical association of Westfalen-Lippe and the University of Muenster (Project identification code: 2018-266-f-S). The study was registered in the German Clinical Trials Register (DRKS00015098).

### 2.2. Study Design and Procedure

The base of the controlled, double-blind intervention study was minimally invasive measurements of glucose and *ß*HB, supported by a determination of caffeine in saliva. Each participant underwent ten interventions (see Table 1), including baseline analytics. Prior to beginning the study, the participants received detailed training on the blood glucose and ketone monitoring system Freestyle Precision Neo from Abbott (Witney, UK) and how to collect the saliva sample with Salivette® from Sarstedt (Nümbrecht, Germany). On the day of the study, participants were asked about their compliance with the fasting time and caffeine abstinence and whether they had acute diseases. They were then assigned to air-conditioned test booths, in which the required test materials (meter, test strips, lancing devices, alcohol swabs, and saliva tubes) were available as well as a computer for the online surveys. The first measurements of capillary blood and implementations of saliva samples were performed immediately after entering the booth. Afterwards, the participants received a test solution in a lidded paper cup. To guarantee blinding, the order of the interventions, preparation of the test solutions, and distribution of the solutions to the participants were performed by different members of the study staff. While ingesting the test solution, the participants were given an online questionnaire about sensory properties. Over a total period of 240 minutes after ingesting the solution, capillary blood measurements and saliva samples were taken at 40-minute intervals. Then, after the 240 minutes of testing, the participants were asked to complete an online questionnaire about hunger and satiety. Furthermore, adverse events were digitally recorded.

**Figure 1:**
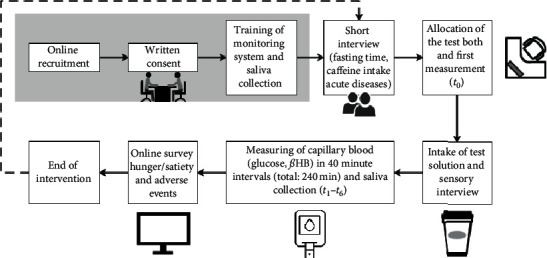
A schematic of the study procedure.

An overview of the study is shown in Figure 1. All participants underwent 10 test days, with different orders of interventions (see Table 1). To maintain a wash-out phase of 24 h, a classical randomisation of the interventions was not possible; thus, the duration of the wash-out phase (24 h) was set according to Fredholm et al. [4]. To guarantee blinding, the order of the interventions was planned individually for each subject by the study management and did not follow the order in Table 1.

### 2.3. Sampling and Analysis

#### 2.3.1. Preparing the Coffee Samples

To prepare the test solutions, decaffeinated coffee (Tchibo; Hamburg, Germany) was made using a coffee machine (Krups; Solingen, Germany). For this process, 50 g ± 1 g of coffee powder was brewed with 1200 mL of water. The beakers were labeled with the participants' IDs and the sample IDs. Depending on the intervention (see Table 1), caffeine (purity: 99.7%; Alfa Aesar; Kandel, Germany), coconut oil (composition: 7.7% C8, 6.0% C10, and 48.5% C12; Vitaquell; Hamburg, Germany), tricaprylin (C8; purity: 99.6%; IOI Oleochemical; Witten, Germany), and/or tricaprin (C10; purity: 99.6%; IOI Oleochemical; Witten, Germany) were weighed into the beakers using an analytical balance (Kern & Sohn; Balingen-Frommern, Germany). Afterwards, 250 mL of decaffeinated coffee was added while stirring, and the beaker was covered with a drinking lid. If MCT was present in the sample solution, 50 mg of soy lecithin (Special Ingredients; Chesterfield, UK) was added for emulsification.

#### 2.3.2. Quantifying the Caffeine with UHPLC

The quantification of the caffeine was performed using a Waters ACQUITY™ Ultra Performance LC with PDA *λ*e Detector and QDA™ Detector, autosampler, in-line degasser, and Waters Empower 3® Software. Chromatographic separation was performed on an RP-18 stationary phase (HSST3, 1.8 *μ*m, 2.1 × 100 mm) at 60°C, using a binary gradient of 0.1% formic acid (A) and acetonitrile/0.1% formic acid (B) at 0.6 mL/min: t0 min 10% B, t5 min 5% B, t3.5 min 20% B, t3.6 min 100% B, t5.6 min 100% B, and t5.7 min 10% B. The detection wavelength was set to *λ* = 272 nm. Unless stated otherwise, ddH_2_O (Millipore®) was used for all UHPLC experiments.

For external standard calibration, 10 mg of reference compound (caffeine, purity: 99.7%; Alfa Aesar; Kandel, Germany) was dissolved in 10 mL of H_2_O, resulting in a concentration of 1 mg/mL. This stock solution was subsequently diluted to 0.5, 0.1, 0.075, 0.05, 0.025, 0.01, and 0.001 mg/mL, and 1 *μ*L of the respective dilutions was injected into UPLC.

The intervention coffee samples were diluted 1 : 10 in H_2_O, and the injection volume for each sample was 1 *μ*L. The quantification for each standard and sample was performed in three independent experiments.

#### 2.3.3. *ß*HB and Glucose in Capillary Blood

Freestyle Precision Neo, test strips, and lancing devices from Abbott (Witney, UK) were used for the capillary blood measurement of glucose and *ß*HB. The participants were trained to perform the measurements and record the results independently on a test protocol. In addition, step-by-step instructions were available in the testing booths.

#### 2.3.4. Caffeine in Saliva

Saliva was collected using Salivette®, in which an absorbent roll is placed in the mouth for 60 seconds, with slight jaw movements but without chewing, and then transferred back into a tube. A timer was provided to ensure proper dwell time. The samples were then stored at −80°C. The caffeine in the saliva was measured at time *t*_0_ on all study days as well as intervention days with test solutions containing caffeine in all samples (*t*_0_–*t*_6_). The determination of caffeine was performed by HPLC (Beckmann Coulter; Krefeld, Germany). To prepare, 50 *μ*L of water, 200 *μ*L of methanol, and 10 *μ*L of a zinc-sulfate solution (30%) were added to 50 *μ*L of saliva obtained after centrifugation. One hundred *μ*L of the supernatant was injected for HPLC analysis. Chromatographic separation was performed isocratically (methanol/water 35 : 65 [v/v]) on a Spherisorb octyl reversed-phase column (5 *μ*m, 4.6 mm × 250 mm) at 0.7 mL/min. The detection wavelength was set to *λ* = 272 nm.

#### 2.3.5. Sensory Interview

An online questionnaire was used to evaluate the taste, aftertaste, mouthfeel, acidity, and caffeine content of the test solution. The participants were asked to rate the solution using a five-point scale and then state whether they believed the drink was caffeine-free (yes/no answer).

#### 2.3.6. Interview on Hunger, Satiety, and Side Effects

At the end of each study day, the participants were asked to complete an online questionnaire consisting of six questions about hunger and satiety during and at the end of the study day. The questionnaire was scored using a 10-point scale, from (1) n*ot at all hungry* to (10) *very hungry* and (1) *not at all satiated* to (10) very *satiated*, for example. In the final question, the participants could indicate positive or negative effects.

### 2.4. Statistical Analysis

The data were prepared using Microsoft Excel 2016 and analysed using IBM SPSS Statistics 24. Kolmogorov–Smirnov test, Shapiro–Wilk test, and graphical analyses were used to evaluate normal distribution. All data were first analysed using descriptive statistics such as minimum, maximum, mean value, median, and standard deviation.

The basis for comparing each intervention was the first capillary blood result from the participants (*t*_0_) to the maximum value (*t*_max_). To calculate the difference between the results, the Wilcoxon test was used. To compare more than two samples, Friedman's two-way analysis of variance (ANOVA; Friedman's test) was performed. The level of significance was *p* < 0.05. For relations among different sample series, the area under the curve (AUC) was calculated for the primary endpoint *ß*HB and the secondary parameter glucose.

To detect a clinically relevant change in the main outcome variable, *ß*HB (mmol/L; *t*_0_ = 0.175; *t*_max_ = 0.329; and SD = 0.065; correlation between *t*_0_ and *t*_max_ = 0; effect size *dz* = 1.5) with a power of 90% and two-sided alpha of 0.05, six participants were required. In case of dropouts, two additional participants (20%; total = 8) were recruited. The sample size was calculated using the scientific freeware G ∗ Power, version 3.1.9.

## 3. Results

Of the 63 applicants, 31 met the inclusion criteria, 9 participated in the consent interview, and 8 were included in the study. One of the participants dropped out on the second day of the study, so the study continued with *n* = 7. The seven healthy adult female participants were 26 ± 3.92 years old and had an average BMI of 21.53 ± 2.05 kg/m^2^.

### 3.1. Caffeine in the Coffee Samples

The solutions containing decaffeinated coffee contained an average of 2.926 ± 0.255 mg of caffeine per 250 mL. Corresponding to the weighed-in amount of caffeine, the mean of the test solutions containing caffeine was 152.790 ± 9.033 mg/250 mL (see Table 1).

### 3.2. *ß*HB and Glucose in Capillary Blood

**Figure 2:**
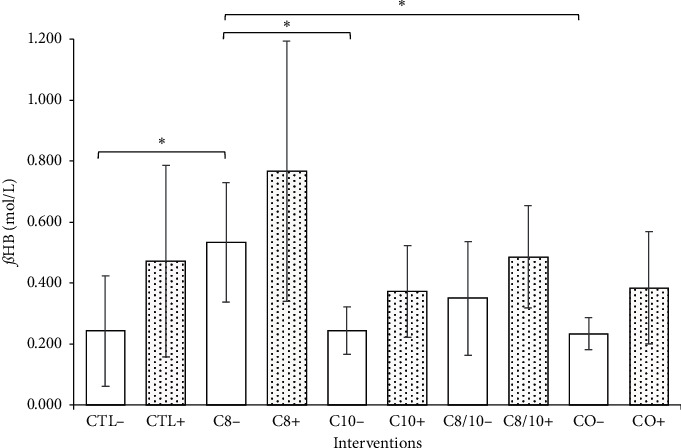
Mean and standard deviation of the *ß*HB concentrations of all interventions. Interventions with caffeine are highlighted. Interventions CTL− vs. C8− (*p*=0.015), C8− vs. C10− (*p*=0.008), and C8− vs. CO− (*p*=0.004) showed significant differences (^*∗*^).

**Figure 3:**
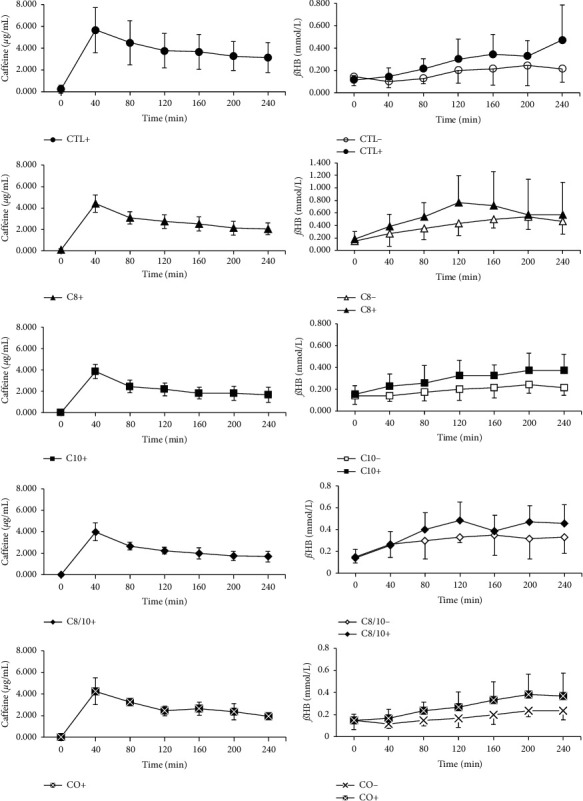
Overview of the curves (mean and standard deviation) of caffeine in saliva (*μ*g/mL) from caffeinated interventions (CTL+, C8+, C10+, C8/10+, and CO+) and of *ß*HB (mmol/L) in capillary blood during all interventions over 240 min (*t*_0_–*t*_6_). The interventions containing caffeine are in black.

The mean of *ß*HB after overnight fasting was 0.148 ± 0.017 mmol/L (min = 0.000; max = 0.400). A significant difference between *t*_0_ and *t*_max_ was shown for all interventions except CTL−, C10−, and CO−; the concentration curves of the caffeinated interventions were consistently above the caffeine-free variants, and the maximum means were significantly higher. No evidence of significant differences between the caffeine-free and the caffeinated interventions was shown using the Friedman test. A significant difference was shown by the interventions CTL− vs. C8− (*p*=.015), C8− vs. C10− (*p*=.008), and C8− vs. CO− (*p*=.004). A difference was observed between C10− vs. C10+ (*p*=.050) and CO− vs. CO+ (*p*=.032) when the AUC was considered. Furthermore, evidence of significance was confirmed for time-concentration curves for the caffeine-free, oil-containing interventions (C8− vs. C10−; C8− vs. CO−). Regardless of statistical testing, interventions with caffeine demonstrated higher concentrations and curve progression of *ß*HB (see Figures 2 and 3).

At the start of the interventions (*t*_0_; overnight fasting), blood glucose levels were within the normal range (87 ± 5.860 mg/dL). The overall course of glucose measurements was unremarkable, and the mean was 84.611 ± 6.678 mg/dL (see Figure S1). No significant differences between the interventions were found.

### 3.3. Caffeine in Saliva

At the beginning of the interventions, salivary caffeine concentrations averaged 0.233 ± 0.367 *μ*g/mL. Accordingly, the instructed caffeine abstinence was observed by the subjects. Forty minutes (*t*_1_) after ingesting the test solutions, the mean maximum of caffeine in saliva was 5.648 ± 2.086 *μ*g/mL. As the interventions progressed, the concentration decreased to 3.123 ± 1.382 *μ*g/mL (240 min). As expected, there were no significant differences between the AUC of the interventions. Compared to the *ß*HB curves, there was no concentration dependence on caffeine, as there were 80–200 minutes between the maxima, depending on the intervention (see Figure 3).

### 3.4. Sensory Interview

The average taste of the solutions was rated on as “neither good nor bad.” There were no noticeable differences between the individual beverages independent of oil or caffeine content. The acidity was rated as “too intense.” The aroma was rated as “just right” or “not intense enough.” Bitterness was also consistently rated as average. In all samples, aftertaste was frequently rated as “too intense” irrespective of caffeine or MCT was added. An oily mouthfeel was rarely noted by the participants in the samples containing MCTs. Further, the caffeine-free interventions could not be distinguished from the caffeinated interventions. In 61% of the interventions, it was indicated by the participants that the sample contained caffeine.

### 3.5. Hunger and Satiety

Due to the overnight fasting, at the beginning of the intervention, the participants were hungry (min 3 ± 3.1 C10+; max 5.29 ± 2.98 C8−). All interventions showed an increase in hunger (min 6.17 ± 1.84 C10+; max 8.43 ± 1.13 C8−) and a decrease in satiety. At the beginning and end of the interventions, all values for satiety were below 3.

### 3.6. Side Effects

The most common side effects of the interventions were difficulty concentrating [9], abdominal pain [9], nausea [7], and headache [7] (see Table 2). The abdominal symptoms were most common in the oil-based interventions. Overall, the caffeine-free interventions tended more to cause side effects.

## 4. Discussion

As expected, only small amounts of caffeine were present in caffeine-free coffee (2.926 ± 0.255 mg/250 mL). When the quality of the caffeine weights was verified, minor deviations (152.790 ± 9.033 mg/250 mL) were present, indicating a uniform intervention course. The use of 150 mg of caffeine per 250 mL of liquid corresponds to the average caffeine content of a regular cup of coffee, or 50–100 mg/150 mL. Based on the test participants, this corresponds to an intake of approx. 2.5 mg caffeine per kg body weight.

The measurements of *ß*HB after overnight fasting were consistent with the data in the literature, which range from 0.07 mmol/L to 0.27 mmol/L [19, 28, 29]. The control without caffeine (CTL−) showed no significant difference between *t*_0_ and *t*_max_. Therefore, there was no significant effect of prolonged food abstinence on *ß*HB concentration during the intervention period. In contrast to the caffeine-free solutions with MCT (C8−, C10−, C8/10−, and CO−), all caffeinated solutions with MCT (C8+, C10+, C8/10+, and CO+) showed a significant increase in *ß*HB between *t*_0_ and *t*_max_. There were only limited indications of significance when comparing between interventions (*ß*HB: CTL− vs. C8−, C8− vs. C10−, C8− vs. CO−; AUC_*ß*HB_: C10− vs. C10+, CO− vs. CO+). All caffeinated interventions showed higher concentrations of *ß*HB than the caffeine-free equivalents, suggesting an additive effect of caffeine. The present high standard deviations of *ß*HB are generally known and correspond to the literature [28, 30]. In summary, the use of C8 resulted in higher levels of *ß*HB in blood. Regardless of statistical testing, the caffeinated interventions demonstrated higher concentrations and curve progressions of ßHB (Figure 3).

In 2017, Vandenberghe et al. investigated the influence of caffeine on ketogenesis. Depending on the caffeine concentration (2.5–5 mg/kg BW), an increase of *ß*HB in plasma of 88–116% was observed [31]. These results are not transferable to the results reported in this study for several reasons. The first is that Vandenberghe et al. administered caffeine dissolved in juice in addition to a breakfast rich in rapidly available carbohydrates. Accordingly, the glucose metabolism (blood glucose, insulin) was influenced, impairing ketogenesis. In addition to this initial disruption of ketogenesis, the rapid metabolism of the meal is likely to result in a catabolic metabolic state later in the intervention, resulting in an increase in glucagon, epinephrine, free fatty acids, and, possibly, *ß*HB [32]. In the present study, blood glucose was stable and showed no abnormalities. Furthermore, a caffeine dosage per kg body weight is not comparable to a standardised, weight-independent administration or typical caffeine consumption (e.g., a coffee-containing drink). However, caffeine intake is associated with increased catabolic hormones, increased lipolysis, and the mobilisation of free fatty acids [10–13]. Thus, the increase in ketogenesis is likely due to the combination of the mechanisms above.

Another study by Vandenberghe et al. showed that intake of C8, followed by a mixture of C8/C10 (60 : 40), exhibited the highest ketogenic potential. With C10 and coconut oil, only minor differences in ketone body concentration were shown compared to the control group [33]. Croteau et al. showed that, after an intake of 30 g of a mixture of C8/C10 or C8 over four weeks, *ß*HB increased by 116% and 129% in mildly affected Alzheimer's patients [34]. Despite the differences in study design, these findings may be confirmed in the present study, as explanations of the properties of MCTs may be an increased binding tendency of C10 to plasma albumins, higher dependence of C10 on CPT1 for transport into the mitochondria, and a higher affinity of acyl-CoA dehydrogenase for C8 [35–37]. In human astrocytes, C8 leads to a stimulation of ketogenesis, and, in contrast, C10 stimulates glycolysis (lactate) [38]. McAllister et al. also found a significant increase in *ß*HB by adding MCTs (28 g and 42 g) to coffee; however, there was no significant difference between the interventions. A standardised dosage of caffeine and mixed MCTs (75% MCT oil of unclear composition and 25% coconut oil) were used, making a direct comparison with the results presented in the present paper impossible [39]. Based on the compositions of the interventions in this study, ketogenicity decreases with increasing chain length.

To avoid additional caffeine consumption, a 24-hour caffeine restriction was implemented, and the caffeine in the participants' saliva was measured as a control. After 24 h of caffeine restriction, the body has metabolised and excreted previously ingested caffeine [4]. The measurement of caffeine in saliva is considered a reliable parameter for plasma caffeine levels. There was no significant difference between the caffeine levels in saliva and plasma, making this minimally invasive procedure suitable [40]. Given the low baseline levels of caffeine in saliva (0.233 ± 0.367 *μ*g/mL; min 0.000; max 0.833), it is reasonable to assume that the participants adhered to the instructions on abstinence. For all interventions, the maximum caffeine concentration in saliva was seen after 40 min (*t*_1_), consistent with known data describing a maximum at 15–120 min after ingestion [4]. Maximum *ß*HB concentrations occurred with an 80–200 min time delay to the maximum caffeine concentration. This delay is plausible based on the assumption that caffeine does not directly stimulate ketogenesis rather activates lipolysis and the release of free fatty acids with subsequent *ß*-oxidation.

The consistent sensory evaluation and misinterpretation of the caffeine content of the test beverages showed that neither the addition of caffeine (strongly bitter taste) nor of MCTs could be detected by the participants. Accordingly, no influence of caffeine or MCTs on the overall taste was observed in the present blind study.

There was no evidence of a reduction in hunger or a positive effect on satiety from intaking MCTs or the stimulation of ketogenesis. Accordingly, the frequently described reduction of the feeling of hunger by *ß*HB could not be verified [27, 41, 42]. There were also no other positive effects, such as more energy or activity, as a result of ingesting the test solutions. The described effects of the “bulletproof coffee” could therefore not be confirmed. Further, undesirable side effects occurred, some of which were due to the unusual conditions (i.e., temporary abstinence from food and caffeine). This explains mild caffeine withdrawal symptoms, such as headache or fatigue, and pseudohypoglycaemia (shakiness, weakness) [4, 43]. Gastrointestinal discomfort after ingesting MCTs is a well-known side effect [44, 45]. In this study, a moderate amount of MCTs was used to avoid specific side effects; however, low doses of MCTs also cause lower ketone-body synthesis and may therefore influence hunger-satiety behaviour.

This study demonstrates that a minimally invasive design for performing simple kinetics studies is possible; however, an intensive introduction and high compliance of participants are necessary. Due to the high number of measurements and the associated time requirement, intensive support of the study participants is essential.

Limitations of this study include a small sample, an underestimation of the total ketone body concentration, measurement errors due to self-measurement by the participants, and that the participants were not blinded to the measurement results. However, the sample size was sufficient to answer the research question. Furthermore, *ß*HB is the main ketone body, and the participants were well trained in measurement performance, which minimised the measurement errors. The blinding can be considered assured, especially as the glucose values were consistently within the normal range. Still, further metabolic knowledge would be necessary to interpret deeper correlations (e.g., side effects, hunger, and satiety). For a deeper understanding of metabolic processes, the study design was inadequate, and measurements such as acetoacetate, free fatty acids, and hormones (insulin and glucagon) are necessary.

## 5. Conclusion

The minimally invasive study design was well suited to obtain an overview of the influence of caffeine and MCTs on ketogenesis. The results indicate that caffeine promotes ketogenesis and that, for MCTs, the influence strongly depends on chain length and the composition of the used fat. A positive effect on hunger and satiety could not be detected. The health benefits of the food trend “bulletproof coffee” and the use of MCTs of different chain lengths and caffeine in clinical diets require further investigation.

## Figures and Tables

**Table 1:** A description of the test solutions.

Intervention	Ingredients of the test solution
CTL−	Decaffeinated coffee
CTL+	Decaffeinated coffee, 150 mg caffeine
C8−	Decaffeinated coffee, 10 g C8
C8+	Decaffeinated coffee, 10 g C8, 150 mg caffeine
C10−	Decaffeinated coffee, 10 g C10
C10+	Decaffeinated coffee, 10 g C10, 150 mg caffeine
C8/10−	Decaffeinated coffee, 5 g C8, 5 g C10
C8/10+	Decaffeinated coffee, 5 g C8, 5 g C10, 150 mg caffeine
CO−	Decaffeinated coffee, 10 g coconut oil
CO+	Decaffeinated coffee, 10 g coconut oil, 150 mg caffeine

− = without caffeine, + = with caffeine, CTL = control, C8 = tricaprylin, C10 = tricaprin, C8/10 = mixture of C8 and C10, and CO = coconut oil.

**Table 2:** The frequency of side effects in all interventions.

Side effects	Interventions									
	CTL−	CTL+	C8−	C8+	C10−	C10+	C8/10−	C8/10+	CO−	CO+
Discomfort	—	1	1	—	—	—	—	1	—	—
Abdominal pain	—	—	2	1	2	—	2	1	—	1
Diarrhea	—	—	1	—	—	—	—	—	—	—
Nausea	1	—	2	—	1	—	1	—	—	2
Difficulty concentrating	1	1	1	1	3	—	—	—	1	1
Headache	3	1	—	1	1	—	—	—	1	—
Fatigue	1	—	—	—	1	—	—	—	2	—
Weakness	—	—	1	—	—	—	—	—	1	—
Dizziness, circulatory problems	—	—	1	—	1	—	—	—	1	—
Shakiness	—	1	—	1	—	—	—	—	—	1

Total	5	4	9	4	9	0	3	2	5	5

Multiple mentions by the participants were possible.

## Data Availability

The data used to support the findings of this study are available from the corresponding author upon request.

## Supplementary Materials

Supplementary Materials Figure S1. The results of the glucose measurements of all interventions.
