# Supplementary material for: Short-Term Influence of Caffeine and Medium-Chain Triglycerides on Ketogenesis: A Controlled Double-Blind Intervention Study
Source: J Nutr Metab. 2021 Jun 15;2021:1861567. doi: 10.1155/2021/1861567 (PMC8221889; doi:10.1155/2021/1861567)
Supplement: Supplementary Materials — Figure S1. The results of the glucose measurements of all interventions. [file 1861567.f1.docx]

Supplementary Material

**Figure S1.** Overview of the curves (mean, standard deviation) of glucose (mg/dL) in capillary blood during all interventions over the total duration of 240 min (t_0_ - t_6_). The interventions containing caffeine are each marked up black.
